# Relationship between aspirin use of esophageal, gastric and colorectal cancer patient survival: a meta-analysis

**DOI:** 10.1186/s12885-020-07117-4

**Published:** 2020-07-09

**Authors:** Ju-Li Lin, Jian-Xian Lin, Chao-Hui Zheng, Ping Li, Jian-Wei Xie, Jia-bin Wang, Jun Lu, Qi-Yue Chen, Long-long Cao, Mi Lin, Chang-Ming Huang

**Affiliations:** 1 Department of Gastric Surgery, Fujian Medical University Union Hospital, No.29 Xinquan Road, Fuzhou, 350001 Fujian Province China; 2 Department of General Surgery, Fujian Medical University Union Hospital, Fuzhou, Fujian Province China; 3 Key Laboratory of Ministry of Education of Gastrointestinal Cancer, Fujian Medical University, Fuzhou, Fujian Province China

**Keywords:** Aspirin, Esophageal cancer, Gastric cancer, Colorectal cancer, Long-term survival

## Abstract

**Background:**

Many studies have found that use of aspirin can lengthen survival in patients with gastrointestinal cancer. The aim of this study was to assess the survival benefit of aspirin use compared with non-aspirin use for patients with esophageal, gastric or colorectal cancer.

**Methods:**

We searched online databases, including PubMed, the Cochrane Library, Embase and www.clinicaltrials.gov for studies that were conducted, before April 30th, 2020, to identify relevant studies. Overall survival and cancer-specific survival of esophageal, gastric and colorectal cancers among aspirin users were compared with those among non-aspirin users. Data extraction and quality evaluation were independently conducted by 2 investigators. A meta-analysis was performed to calculate the pooled risk ratios (RRs) for overall survival and cancer-specific survival by using either a fixed-effects model or a random-effects model.

**Results:**

A total of 18 studies were included in this meta-analysis, with more than 74,936 patients. There were no significant differences between postdiagnosis aspirin use and overall survival for esophageal and gastric cancers. For colorectal cancer, a benefit that was associated with postdiagnosis aspirin use was observed for overall survival and cancer-specific survival [HR = 0.83, 95%CI(0.75, 0.9.);HR = 0.78, 95%CI(0.66, 0.92), respectively. However, a prediagnosis of aspirin use did not provide a benefit for overall or cancer-specific survival in colorectal cancer. HR values for overall and cancer-specific survival benefits for colorectal cancer associated with both prediagnosis and postdiagnosis aspirin were as follows: HR = 0.75, 95%CI(0.61, 0.92) and HR = 0.78, 95%CI(0.73, 0.85), respectively. In addition, the survival benefit of postdiagnosis aspirin use appeared to be confined to patients with mutated PIK3CA tumors [HR = 0.78, 95%CI(0.50, 0.99)] and was positive for PTGS2 (COX-2) expression [HR = 0.75, 95%CI(0.43, 1.30)].

**Conclusions:**

These findings provide further indications that postdiagnosis aspirin use improves overall survival and cancer-specific survival in colorectal cancer, especially for patients who are positive for PTGS2 (COX-2) expression and PIK3CA-mutated tumors. However, aspirin therapy does not improve overall survival in esophageal and gastric cancers, although the meta-analysis was mainly limited to retrospective studies.

## Background

Esophageal, gastric and colorectal cancers are the most common cancers of the digestive tract [1]. Many factors, including old age and poor living habits, are risk factors for gastrointestinal malignancies. Although the incidence and mortality of gastrointestinal malignancies have been reduced in recent years, the comprehensive treatment of gastrointestinal malignancies has progressed slowly in recent decades. Therefore, it is urgent to discover a more effective comprehensive treatment for gastrointestinal malignancies. Aspirin is a nonselective cyclooxygenase inhibitor with strong antipyretic and analgesic effects and is widely used for its anti-inflammatory and anti-rheumatic properties. For example, small doses of aspirin are used to prevent the onset of cardiovascular disease, cerebrovascular disease and transient ischemic attacks. In recent years, many studies [2–7] have found that aspirin also has anticancer effects. However, as there are still some controversy about these studies, the aim of this study was to assess the survival benefits of aspirin use (compared with non-aspirin use) for esophageal, gastric and colorectal cancer patients through the use of a meta-analysis.

## Methods

All of the search results were evaluated according to the Preferred Reporting Items for Systematic Reviews and Meta-Analyses (PRISMA 2009) statement [8].

### Inclusion and exclusion criteria

The inclusion criteria were as follows: (1) RCTs or observational studies including cohort and case-control studies; (2) the outcomes of interest beingdefined as OS (overall survival) and CSS (cancer-specific survival) of esophageal, gastric, colorectal, colon or rectal cancer; (3) the study addressing aspirin usage at the times of prediagnosis and/or postdiagnosis of esophageal, gastric, colorectal, colon or rectal cancer; (4) HR or OR estimates with 95% CIs were available. The exclusion criteria were as follows: (1) duplicate articles; (2) inadequate data; and (3) sample sizes less than 20; (4) NOS ≤5.

### Literature search

We conducted a comprehensive systematic literature search of online databases, including PubMed, the Cochrane Library, Embase and www.clinicaltrials.gov for studies that were conducted before April 30th, 2020, to identify all RCTs and observational studies. The following key words were used in these literature searches: (‘colorectal cancer’ or ‘colon cancer’ or ‘rectal cancer’ or ‘colorectal adenocarcinoma’ or ‘colon adenocarcinoma’ or ‘rectal adenocarcinoma’) AND (‘aspirin’ or ‘non-steroidal anti-inflammatory drugs’ or ‘NSAIDS’) (‘gastric cancer’ or ‘gastric adenocarcinoma’) AND (‘aspirin’ or ‘non-steroidal anti-inflammatory drugs’ or ‘NSAIDS’) (‘esophageal cancer’ or ‘esophageal adenocarcinoma’ or ‘esophageal squamous cell carcinoma’) AND (‘aspirin’ or ‘non-steroidal anti-inflammatory drugs’ or ‘NSAIDS’). There were no language restrictions. We also reviewed the references of the included articles and of the related systematic reviews, in order to identify additional studies.

### Study selection and quality assessment

The qualities of the included non-RCTs were assessed by using the Newcastle–Ottawa Scale (NOS) [9]. The scale utilizes a score system ranging from 0 to 9, and the quality of the observational studies were considered to be high-quality with a score of 5 or higher.

### Data extraction

Data extraction and the evaluation of the quality of the literature were independently conducted by 2 investigators (Ju-li Lin and Jian-xian Lin). At time when there was any uncertainty about the inclusion of a study, the issue was discussed between the two investigators to achieve a resolution. A Microsoft Excel database was employed to record all of the available information, including the baseline details, title, first author’s name, year of publication, study design, region, journal, sample size, period of patient recruitment, follow-up time, and HRs.

### Statistical analysis

The Cochran’s Q statistic and I^2^ statistics were applied to assess the heterogeneity among all of the studies [10]. For the Q statistic, a *p* value of less than 0.1 was considered to be statistically significant. When statistical heterogeneity was detected, the sources of the heterogeneity were explored, and sensitivity analyses were performed. A random-effects model was used if heterogeneity existed; otherwise, the fixed-effect model was used. When possible, subgroup analyses were conducted to assess the potential impacts of the mutation statuses. The cut-off point for quality among observational studies (NOS ≤5 vs. NOS > 5) was arbitrarily defined. Publication bias was assessed using the Begg and Egger regression asymmetry test, together with funnel plots. All of the statistical analyses were conducted by using STATA, version 13.0 (Stata Corporation, College Station, TX).

## Results

### Retrieved studies and characteristics

According to the previously described search strategy, 3612 citations were obtained from the online database up until April 30th, 2020. A total of 3569 articles were excluded by viewing the titles and abstracts. The full texts of 36 records were read. Ultimately, 18 full-text studies [4–7, 11–24] were obtained and assessed according to the eligibility criteria, including 1 case-control study and 17 cohort studies, with the studies comprised of more than 74,936 patients. The detailed literature search and screening process are shown in Supplement Figure 1. The characteristics included in the study are shown in Tables 1 and 2, including the first author’s name, year of publication, study design, region, journal, sample size, period of patient recruitment patients, follow-up time and definition of aspirin use.

**Table 1:** Characteristics of the included trials and particiants

Included Trials	Dedign	Region	database	Journal	Sample size	Period	aspirin use	Follow-up time	Surgery^a^ non-user/ user	Chemotherapy^b^ non-user/ user
gastric cancer										
Spence et al. [11] 2018	Cohort study	United Kingdom	cancer registries in England	Gastroenterology	2391	1998–2012	post-diagnosis use	until September 2015	947 (50.0%) / 273 (55.0%)	720 (38.0%) / 146 (29.4%)
Spence et al. [11] 2018	Cohort study	United Kingdom	the Scottish Cancer Registry	Gastroenterology	1442	2009–2012	post-diagnosis use	until January 2015	376 (33.3%) / 124 (39.7%)	587 (51.9%) / 145 (46.5%)
Frouws et al. [7] 2017	Cohort study	Netherlands	Eindhoven Cancer Registry	British Journal of Cancer	750	Jan 1998-Dec 2011	Pre- and post-diagnosis use	NA	Unknown	Unknown
esophageal cancer										
Macfarlane et al. [13] 2015	Cohort study	United Kingdom	PCCIU databaseIN Scotland	Cancer Epidemiology	1197	1996–2010	Pre- and post-diagnosis use	9	Unknown	Unknown
Spence et al. [11] 2018	Cohort study	United Kingdom	cancer registries in England	Gastroenterology	2733	1998–2012	post-diagnosis use	until September 2015	879 (40.4%) 215(38.5%)	108(50.0%) 235(42.0%)
Spence et al. [11] 2018	Cohort study	United Kingdom	the Scottish Cancer Registry	Gastroenterology	1921	2009–2012	post-diagnosis use	until January 2015	266 (18.5%) 90 (18.6%)	883 (61.5%) 256 (52.8%)
Frouws et al. [7] 2017	Cohort study	Netherlands	Eindhoven Cancer Registry	British Journal of Cancer	946	Jan 1998-Dec 2011	Pre- and post-diagnosis use	NA	Unknown	Unknown
Colorectal cancer										
Chan et al. [17] 2009	Cohort study	USA	Nurses’ Health Study and the Health Professionals Follow-up Study	JAMA	1279	1980–2002	Pre- and post-diagnosis use	11.8 years	Unknown	Unknown
Liao et all [20]. 2012	Cohort study	USA	Nurses’ Health Study and Health Professionals Follow-up Study	NEJM	964	1976-July 1st2006	Pre- and post-diagnosis use	until death or January 2011	Unknown	Unknown
Walker et al. [20] 2012	Cohort study	UK	General Practice Research Database	British Journal of Cancer	13,994	1987–2010	Pre- and post-diagnosis use	1.7–3.1 years	Unknown	Unknown
Domingo et al. [18] 2013	Cohort study	UK	VICTOR trial	J Clin Oncol	896	Apr 2002-Sep 2004	post-diagnosis use	NA	All patients	430 (63.1%) 62 (55.9%) 59(65.6%)7 (50%)
McCowan et al. [19] 2013	Cohort study	Tayside, United Kingdom	Health Informatics Centre	European Journal of Cancer	2990	1st January 1997-30th December 2006	Pre- and post-diagnosis use	2.8 years	Unknown	Unknown
Kothari et al. [21] 2015	Cohort study	Australia and USA	Moffitt Cancer Center and Royal Melbourne Hospital	Acta Oncol	1487	1996–2010	post-diagnosis use	4.5 years	All patients	Unknown
Reimers et al. [5] 2014	Cohort study	Netherlands	Eindhoven Cancer Registry	JAMA Intern Med.	999	2002–2008	post-diagnosis use	until January 1, 2012or death	All patients	Unknown
Cardwell et al. [16] 2014	Case-control	UK	National Cancer Data Repository	Gastroenterology	4794	1998–2007	Pre- and post-diagnosis use	7.2 years	Unknown	Unknown
Bains et al. [6] 2016	Cohort study	Norway	Cancer Registry of Norway	J Clin Oncol	23,162	Jan 2004-Dec 2011	Pre- and post-diagnosis use	median 3.0 yearsafter CRC diagnosis	88.9% of the patients	Unknown
Frouws et al. [7] 2017	Cohort study	Netherlands	Eindhoven Cancer Registry	British Journal of Cancer	6335	Jan 1998-Dec 2011	Pre- and post-diagnosis use	NA	Unknown	Unknown
Newcomb et al. [14] 2017	Cohort study	USA, Canada,Australia	Four database	J Clin Oncol	2419	1997–2008	Pre- and post-diagnosis use	10.8 years	Unknown	Unknown
Gray et al. [23] 2018	Cohort study	UK	Scottish Cancer Registry	BMC Cancer	8391	Jan 2009 - Jan2015	Pre- and post-diagnosis use	3.6 years	2167 (34.7%) 472 (22.0%)	5908 (94.7%) 2020 (94.0%)
Joseph et al. [24] 2019	Cohort study	Hong Kong	Hong Kong Hospital	J Gastroenterol Hepatol	3292	2004–2015	post-diagnosis use	10 years	All received surgery	Unknown
Zell et al. [15] 2009	Cohort study	USA	California Teachers Study cohort	Cancer	621	Date of diagnosis to death or to December 31, 2005.	Pre-diagnosis use	2.8 years	26 (7%) 19 (8%)	361 (91%) 207 (92%)
Din et al. [4] 2010	Case–control study	UK	Study of Colorectal Cancer in Scotland	Gut	4080	to 30 April 2008	Pre-diagnosis use	NA	Unknown	Unknown
Coghill et al. [14] 2011	Cohort study	USA	Hutchinson Cancer Research Center AND SEER	Gut	1737	1997–2002	Pre-diagnosis use	8 years	Unknown	Unknown

^a^: 947 (50.0%) /273 (55.0%) means 947 (50.0%) receive surgery in aspirin non-user patients and 273 (55.0%) receive surgery in aspirin user patients

^b^: 720 (38.0%) /146 (29.4%) means 720 (38.0%) receive chemotherapy in aspirin non-user patients and 146 (29.4%) receive chemotherapy in aspirin user patients

**Table 2:** Characteristics of the included trials and particiants

Included Trials	Stage^a^ non-user/ user	Dosage	Duration	Reason	Outcomes
gastric cancer					
Spence et al. [11] 2018	I 28 (1.5%) 12 (2.4%) II 43 (2.3%) 20 (4.0%) III 59 (3.1%) 16 (3.2%) IV 119 (6.3%) 16 (3.2%) Missing 1646 (86.9%) 432 (87.1%)	Low-dose aspirin (75 mg) use	182, 365, 548 and 730 tablets	Unknown	not associated with increased survival in sophageal or gastric cancer
Spence et al. [11] 2018	Unknown	Low-dose aspirin (75 mg) use	182, 365, 548 and 730 tablets	Unknown	not associated with increased survival in sophageal or gastric cancer
Frouws et al. [7] 2017	Unknown	Nonusers were defined as patients who received for less than 30 days or never used aspirin.	Unknown	Unknown	increased survival in cancers
esophageal cancer					
Macfarlane et al. [13] 2015	Unknown	Unknown	Unknown	Unknown	improved survival was observed
Spence et al. [11] 2018	I 34 (1.6%) 10 (1.8%) II 69 (3.2%) 28 (5.0%) III 183 (8.4%) 47 (8.4%) IV 132 (6.1%) 23 (4.1%) Unknown 1756 (80.8%) 451 (80.7%)	Low-dose aspirin (75 mg) use	182, 365, 548 and 730 tablets	Unknown	not associated with increased survival in sophageal or gastric cancer
Spence et al. [11] 2018	Unknown	Low-dose aspirin (75 mg) use	182, 365, 548 and 730 tablets	Unknown	not associated with increased survival in sophageal or gastric cancer
Frouws et al. [7] 2017	Unknown	Nonusers were defined as patients who received for less than 30 days or never used aspirin.	Unknown	Unknown	increased survival in cancers
Colorectal cancer					
Chan et al. [17] 2009	I 228 (32%) 193 (35%) II 260 (36%) 186 (33%) III 231 (32%) 181 (32%) I 218 (30%) 203 (37%) II265 (36%) 181 (33%) III 247 (34%) 165 (30%)	used aspirin 2 or more timesper week	Unknown	Headache, arthritis and other musculoskeletal pain, cardiovascular disease	associated with lower risk of colorectal cancer–specific and overall mortality
Liao et all [20]. 2012	I 112 (24%) 102 (30%) II 159 (34%) 87 (26%) III 128 (27%) 99 (29%) IV 31 (7%) 18 (5%) Unknown 36 (8%) 31 (9%) I 19 (20%) 27 (41%) II 36 (38%) 19 (29%) III 23 (24%) 14 (21%) IV 12 (13%) 3 (5%) Unknown 5 (5%) 3 (5%)	as regular use of aspirin duringmost weeks	Unknown	Headache, arthritis and other musculoskeletal pain, cardiovascular disease	associated with longer survival among patients with mutated-PIK3CA colorectal cancer
Walker et al. [20] 2012	Unknown	a repeat prescription (> 2) within the period	a fixed period of 1 year post-diagnosis	Unknown	have a potential as anti-neoplastics in diagnosed colorectal cancer
Domingo et al. [18] 2013	II 332 (48.7%) 57 (51.4%) III 349 (51.2%) (54 48.6%) II 46 (51.1%) 8 (57.1%) III 44 (48.9%) 6 (42.9%)	taking regularlow-dose aspirin at random assignment or who started during follow-up	Unknown	adjuvant setting of colorectal cancer:	support the prospective evaluation of adjuvant low-dose aspirin in patients with tumor PIK3CA mutation
McCowan et al. [19] 2013	Unknown	28 tablets at one per day gave coverage for that prescription of 28 days.	date of the first prescription post-diagnosis to the end of coverage of the last prescription	Unknown	use post-diagnosis of colorectal cancer may reduce both all cause and colorectal cancer specific mortality
Kothari et al. [21] 2015	I 6(4%) 2(4%) II 50(37%) 16(33%) III 45(33%) 22(45%) IV 35(26%) 9(18%)	at least 75 mg of aspirin daily at the time of CRC diagnosis	Unknown	Unknown	significant improvements in survival in PIK3CA-mutated CRC patients
Reimers et al. [5] 2014	I 95 (13.8%) 38(21.2%) II 218 (31.9%) 69(38.5%) III 219 (32.0%) 57(31.8%) IV149(21.8%) 15(0.8%) Unknown 3 (0.4%)	given a prescription for aspirin for 14 days or more after a colon cancerdiagnosis	Unknown	Unknown	Increased PTGS2 expression or the presence of mutated PIK3CA did not predict benefit from aspirin
Cardwell et al. [16] 2014	I 65 (4.2%) II 283 (18.2%) III 565 (36.2%) IV 187 (12.0%) Missing 459 (29.4%)	low dose if 75 mg(0.3% of prescriptions after cancer diagnosis were 25 mg,98.5% were 75 mg, and 1.2% were 300 mg).	Duration of use was determined from quantity of tablets.	Unknown	low-dose aspirin usage after diagnosis of colorectal cancer did not increase survival time.
Bains et al. [6] 2016	I 3600 (21.9%) 1631 (27.7%) II 4840 (29.4%) 2112 (35.9%) III 4829 (29.3%) 1581 (26.8%) IV 3188 (19.4%) 565 (9.6%)	three or more prescriptions of aspirin starting from 30 days after the diagnosis of CRC	Aspirin prescriptions lasted 3 months at a time (100-tablet packets, one tablet once per day),	Unknown	Aspirin use after the diagnosis of CRC is independently associated with improved CSS and OS.
Frouws et al. [7] 2017	Unknown	Nonusers were defined as patients who received for less than 30 days or never used aspirin.	Unknown	Unknown	increased survival in cancers
Newcomb et al. [14] 2017	I 326 (30%) 311 (36%) II 391 (36%) 259 (30%) III 263 (24%) 225 (26%) IV 106 (10%) 61 7 (%) Unknown 311,166	using the medications at least twice per week for more than 1 month	Pre-diagnostic use 1 year before diagnosis /post-diagnostic use between baseline and the 5-year follow-up interview	Unknown	regular use of NSAIDs after CRC diagnosis was significantly associated with improved survival in individuals with KRAS wild-type tumors
Gray et al. [23] 2018	A 1683(27.0%) 597(27.8%) B 2340(37.5%) 851(39.6%) C 2218(35.5%) 702(32.7%)	Low-dose (75 mg) aspirin exposure was identified from dispensing records within this database	users after a lag of 6 months after their first aspirin prescription	Unknown	either before or after diagnosis, did not prolong survival in this population-based CRC cohort.
Joseph et al. [24] 2019	Unknown	no less than 80 mg per day	at least a month	Unknown	lowers risk of both CRC-related mortality and overall mortality
Zell et al. [15] 2009	Unknown	taken aspirin regularly at least once a week	the total duration of use in number of years (< 1, 1, 2, 3–4, 5–9, or 10).	Unknown	NSAIDs are associated with decreased mortality among female CRC patients
Din et al. [4] 2010	Unknown	reported intake of aspirin	Unknown	Unknown	NSAID use prior to CRC diagnosis does not influence survival of colorectal cancer
Coghill et al. [14] 2011	Unknown	at least twice per week for 1 month	first, 0–6 months; second, 6 monthse2.5 years; third, 2.5–7 years; fourth, > 7 years).	Unknown	regular use of NSAIDs prior to diagnosis is associated with improved colorectal cancer survival

^a^: stage I 28 (1.5%) 12 (2.4%) means 28 (1.5%) are stage I aspirin non-user patients and 12 (2.4%) are stage I aspirin user patients

The qualities of 18 studies was assessed by using NOS; four studies achieved a score of 6, six studies achieved a score of 7 and eight studies achieved a score of 8 (Tables 3 and 4). Thirteen studies stated a clear follow-up time. The longest median follow-up period was 10.8 years. Six studies reported a clear definition of the use of PPIs. Seven studies compared the risk of gastric cancer between PPI users and non-PPI users. Thirteen studies evaluated the association between prediagnosis aspirin use and colorectal cancer survival. Thirteen studies evaluated the association between postdiagnosis aspirin use and colorectal cancer survival.

**Table 3:** Quality assessment of the observational studies using the Newcastle-Ottawa Scale (NOS). Assessment of the cohort studies

Author	year	Representativeness of the exposed cohort	Selection of the non-exposed cohort	Ascertainment of exposure to implants	Demonstration that outcome of interest was not present at start of study	Comparability of cohorts		Assessment of outcome	Was follow up long enough for outcomes to occur	Adequacy of follow up of cohorts	Total score
Chan et al. [17]	2009	+	+	+	+	+	–	+	+	+	8
Coghill et al. [14]	2011	+	+	+	+	+	–	+	+	+	8
Bains et al. [6]	2016	+	+	+	+	+	–	+	+	+	8
Liao et all [20].	2012	+	+	+	+	+	–	+	+	+	8
Walker et al. [20]	2012	+	+	–	–	+	+	+	–	+	6
Domingo et al. [18]	2013	+	+	–	+	+	+	+	–	+	7
Frouws et al. [7]	2017	+	+	–	+	+	+	+	–	+	7
Gray et al. [23]	2018	+	+	–	–	+	–	+	+	+	6
Kothari et al. [21]	2014	+	+	–	–	+	–	+	+	+	6
McCowan et al. [19]	2013	+	+	–	+	+	+	+	–	+	7
Macfarlane et al. [13]	2015	+	+	–	–	+	–	+	+	+	6
Newcomb et al. [14]	2017	+	+	+	+	+	–	+	+	+	8
Reimers et al. [5]	2014	+	+	–	+	+	+	+	–	+	7
Spence et al. [11]	2017	+	+	+	+	+	+	+	–	+	8
Zell et al. [15]	2009	+	+	–	+	+	+	+	–	+	7
Joseph et al. [24]	2019	+	+	–	+	+	+	+	–	+	7

**Table 4:** Quality assessment of the observational studies using the Newcastle-Ottawa Scale (NOS). Assessment of the case–control study

Author	year	Is the case definition adequate	Representativeness of the cases	Selection of Controls	Definition of Controls	Comparability	Ascertainment of exposure	Same method of ascertainment for cases and controls	Non-Response Rate	Total score
Cardwell et al. [16]	2014	+	+	+	+	+	+	+	+	8
Din et al. [4]	2010	+	+	+	+	+	+	+	+	8

### Association between postdiagnosis aspirin use and survival (OS and CSS) in esophageal and gastric cancers

**Fig. 1:**
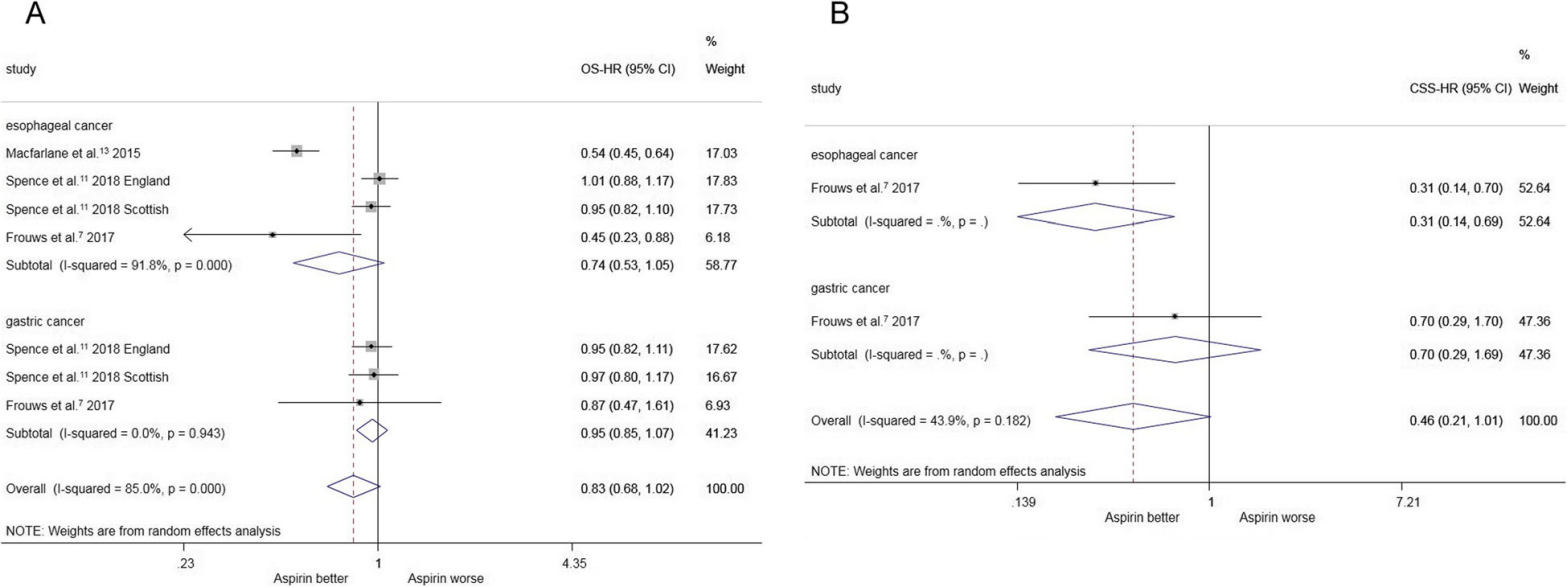
**a** post-diagnosis aspirin use and overall survival for upper digestive cancer. **b** post-diagnosis aspirin use and cancer specific survival for upper digestive cancer

Three studies (involving 6797 patients) compared the overall survival of esophageal cancer among aspirin users compared with non-aspirin users. The estimated pooled HRs showed no significant differences between the two groups [HR = 1.009, 95%CI(0.847, 1.202)] (Fig. 1a).

Two studies (involving 4589 patients) compared the overall survival of gastric cancer among aspirin users compared with non-aspirin users, and the estimated pooled HRs indicated no significant differences between the groups [HR = 0.870, 95%CI(0.470, 1.610)] (Fig. 1a).

Three studies (involving 11,380 patients) compared the overall survival of upper digestive cancer among aspirin users compared with non-aspirin users, with no significant differences between the two groups based on estimated pooled HRs [HR = 0.831, 95%CI(0.679, 1.016)] (Fig. 1a).

One study (involving 946 patients) compared the cancer-specific survival of esophageal cancer among aspirin users with non-aspirin users; based on HRs, the use of aspirin postdiagnosis was associated with longer cancer-specific survival [HR = 0.34, 95%CI(014, 0.69)] (Fig. 1b). One study involving 750 patients compared the cancer-specific survival of gastric cancer among aspirin users with non-aspirin users, and the HRs revealed no significant differences between the groups [HR = 0.70, 95% CI (0.29, 1.69)] (Fig. 1b).

### Association between postdiagnosis aspirin use and survival (OS and CSS) in colorectal cancer

**Fig. 2:**
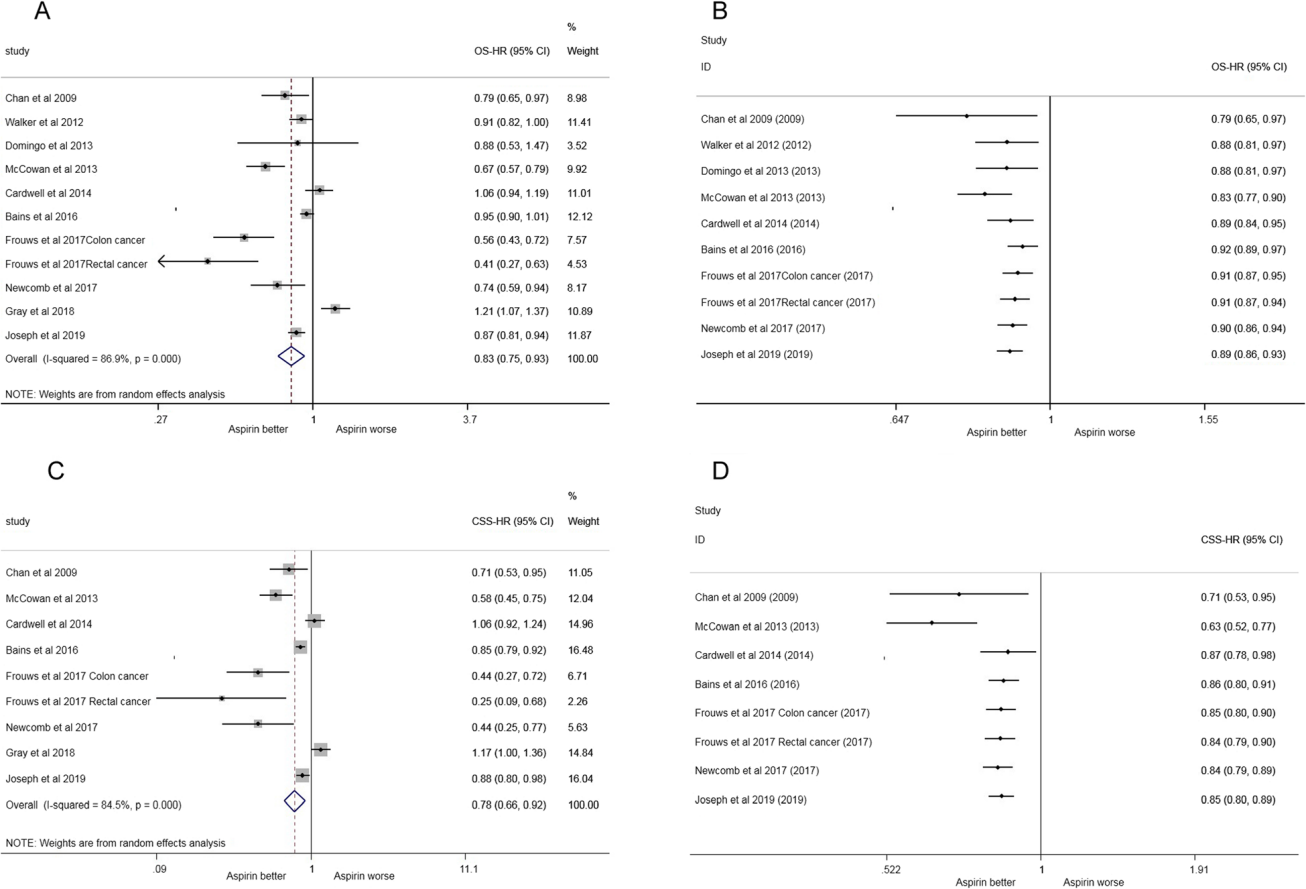
**a** post-diagnosis aspirin use and overall survival for colorectal cancer.**b** cumulative meta-analysis of the HR for the colorectal cancer according to time. **c** post-diagnosis aspirin use and cancer specific survival for colorectal cancer. **d** cumulative meta-analysis of the HR for the colorectal cancer according to time

Ten studies (involving 67,552 patients) compared the overall survival of colorectal cancer among aspirin users compared with non-aspirin users. According to the estimated pooled HRs, the use of aspirin postdiagnosis was associated with longer overall survival [HR = 0.83, 95%CI(0.75, 0.93)] (Fig. 2a).

The result of cumulative meta-analysis showed that the significant difference supporting PPI use was first found in the latest study in Joseph et al. [HR = 0.89, 95% CI(0.86–0.93)], with the CI narrowing and the effect size becoming stable (Fig. 2b).

Eight studies (involving 52,662 patients) compared cancer-specific survival in colorectal cancer among aspirin users and non-aspirin users. The estimated pooled HRs showed that the use of aspirin postdiagnosis was associated with longer overall survival [HR = 0.78, 95%CI(0.66, 0.92)] (Fig. 2c).

The result of cumulative meta-analysis indicated that the significant difference supporting PPI use was first found in the latest study by Joseph et al. [HR = 0.85, 95% CI (0.80–0.89], with the CI narrowing and the effect size becoming stable (Fig. 2d).

### Association between prediagnosis aspirin use and survival (OS and CSS) in colorectal cancer

**Fig. 3:**
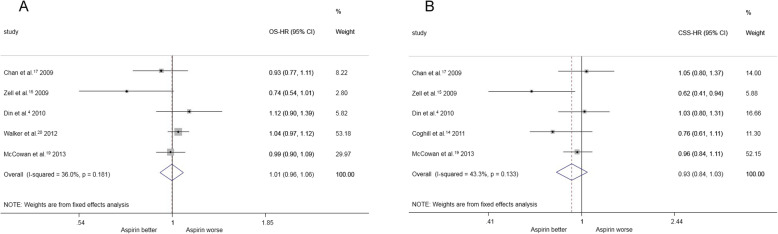
**a** pre-diagnosis aspirin use and overall survival for colorectal cancer. **b** pre-diagnosis aspirin use and cancer specific survival for colorectal cancer

With regard to overall survival in colorectal cancer, five studies involving 6202 patients compared among aspirin users compared with non-aspirin users. The estimated pooled HRs demonstrated no significant differences between the two groups [HR = 1.01, 95%CI(0.96, 1.06)] (Fig. 3a).

Five studies (involving 45,101 patients) compared the cancer-specific survival of colorectal cancer among aspirin users compared with non-aspirin users, and according to the estimated pooled HRs, there were no significant differences between the groups [HR = 0.93, 95%CI(0.84, 1.03)] (Fig. 3b).

### Association between both prediagnosis and postdiagnosis aspirin use and survival (OS and CSS) in colorectal cancer

**Fig. 4:**
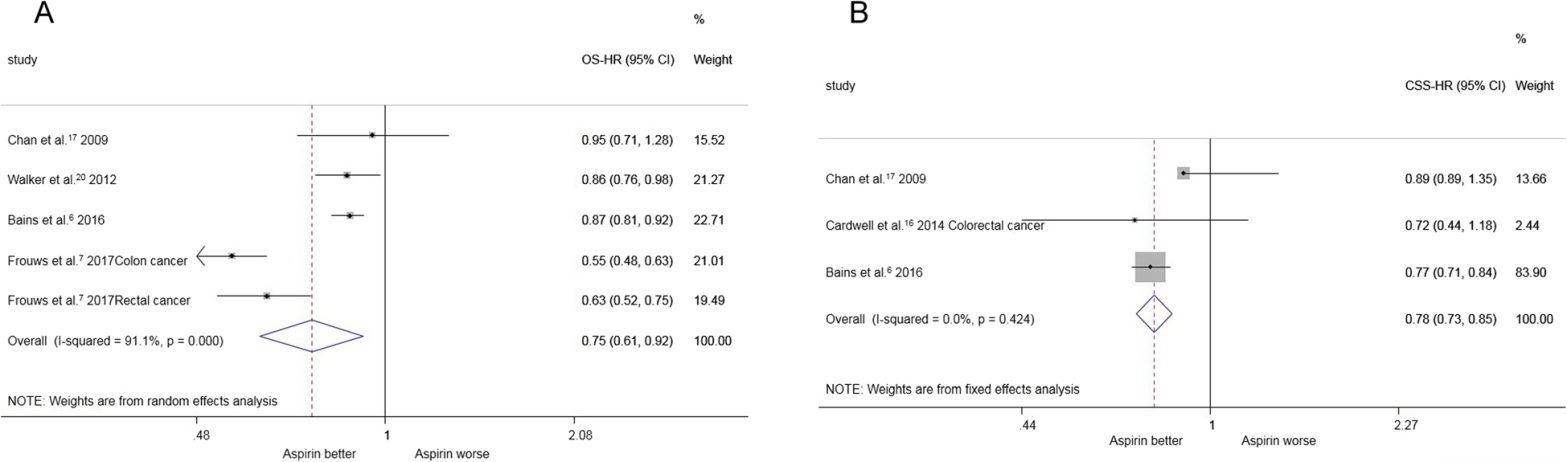
**a** both pre and post-diagnosis aspirin use and overall survival for colorectal cancer. **b** both pre and post -diagnosis aspirin use and cancer specific survival for colorectal cancer

Four studies (involving 2350 patients) compared the overall survival of colorectal cancer among aspirin users compared with non-aspirin users. The estimated pooled HRs revealed that the use of aspirin both prediagnosis and postdiagnosis was associated with longer overall survival [HR = 0.75, 95%CI(0.61, 0.92)] (Fig. 4a).

Three studies (involving 1849 patients) compared cancer-specific survival in colorectal cancer among aspirin users compared with non-aspirin users, and the estimated pooled HRs indicated that the use of aspirin both prediagnosis and postdiagnosis was associated with longer overall survival [HR = 0.78, 95%CI(0.73, 0.85)] (Fig. 4b).

### Subgroup analysis according to the PIK3CA gene status

**Fig. 5:**
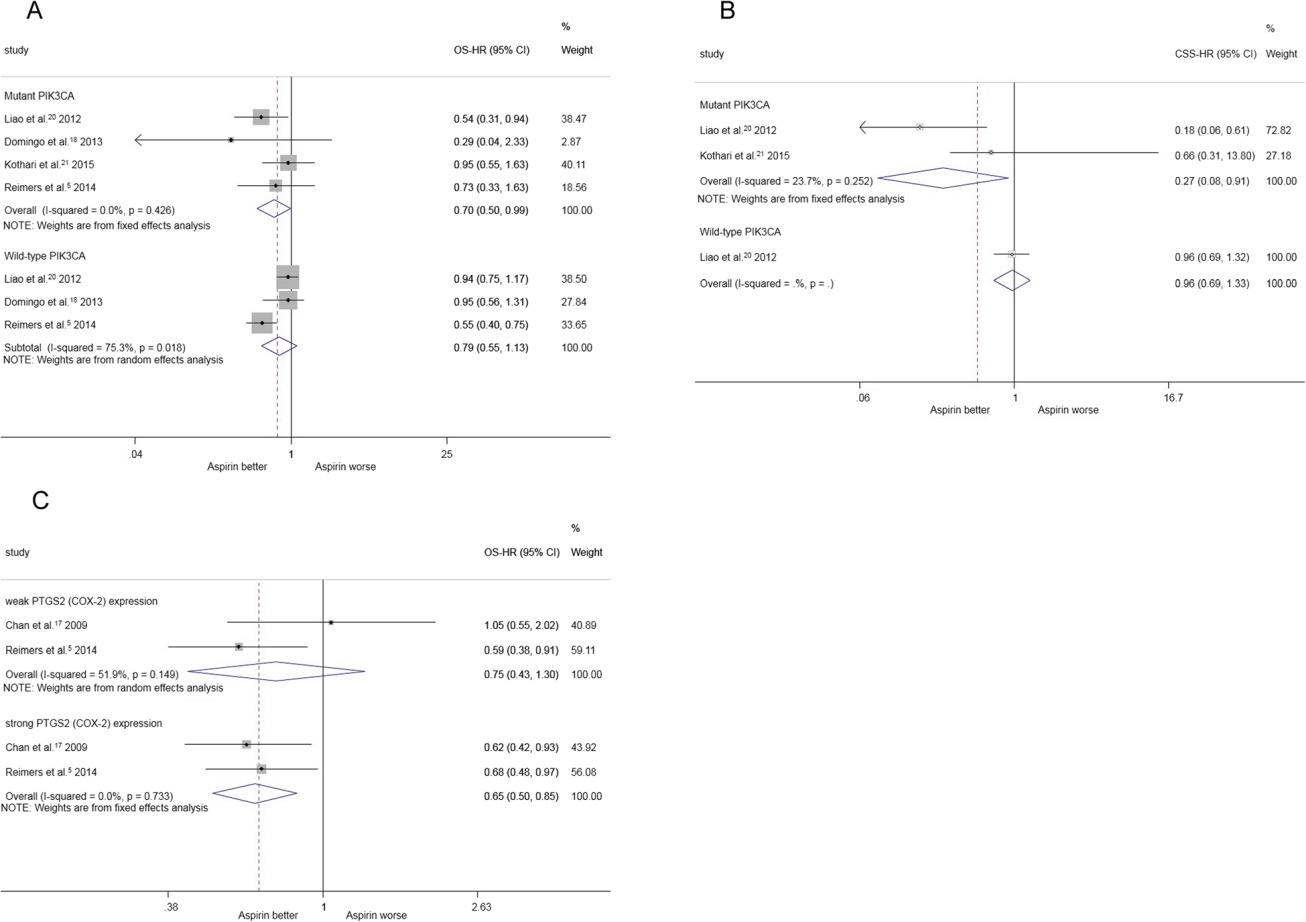
**a** post-diagnosis aspirin use and overall survival for colorectal cancer according to PIK3CA mutation. **b** post-diagnosis aspirin use and cancer specific survival for colorectal cancer according to PIK3CA mutation. **c** post-diagnosis aspirin use and overall survival for colorectal cancer according to PTGS2(COX-2) mutation

Four studies (involving 4346 patients) compared the overall survival of colorectal cancer among aspirin users compared with non-aspirin users among those with PIK3CA gene mutation. Based on the estimated pooled HRs, the use of aspirin postdiagnosis was associated with longer overall survival [HR = 0.70, 95%CI(0.50, 0.99)] (Fig. 5a).

For overall survival in colorectal cancer, three studies involving 8490 patients compared among aspirin users compared with non-aspirin users among patients with a wild-type PIK3CA gene, and the estimated pooled HRs showed no significant differences between the groups [HR = 0.79, 95%CI(0.53, 1.13)] (Fig. 5a).

Two studies involving 2451 patients compared the cancer-specific survival in colorectal cancer among aspirin users compared with non-aspirin users among patients with a mutated PIK3CA gene. The estimated pooled HRs showed that the use of aspirin postdiagnosis was associated with longer overall survival [HR = 0.27, 95%CI(0.08, 0.91)] (Fig. 5b).

### Subgroup analysis according to the PTGS2 (COX-2) expression status

Two studies involving 560 patients compared overall survival in colorectal cancer among aspirin users compared with non-aspirin users in patients with strong PTGS2 (COX-2) expression. According to the estimated pooled HRs, the use of aspirin postdiagnosis was associated with longer overall survival [HR = 0.65, 95%CI(0.54, 0.83)] (Fig. 5c).

Regarding the overall survival of colorectal cancer, two studies involving 4328 patients compared aspirin users with non-aspirin users among patients with weak PTGS2 (COX-2) expression. The estimated pooled HRs showed no significant differences between the two groups [HR = 0.75, 95%CI(0.43, 1.30)] (Fig. 5c).

### Subgroup analysis according tumor stage

Four studies involving 28,032 patients compared overall survival in colorectal cancer among aspirin users compared with non-aspirin users among patients. The estimated pooled HRs showed no significant differences between the groups (Supplement Figure 3A).

Five studies involving 32,826 patients compared cancer specific survival in colorectal cancer among aspirin users compared with non-aspirin users. The estimated pooled HRs showed no significant differences between the groups in stage I, stage III and stage IV patients. While the use of aspirin was associated with longer cancer specific survival in stage II patients [HR = 0.65, 95%CI(0.54, 0.83)] (Supplement Figure 3B).

### Sensitivity analysis

**Fig. 6:**
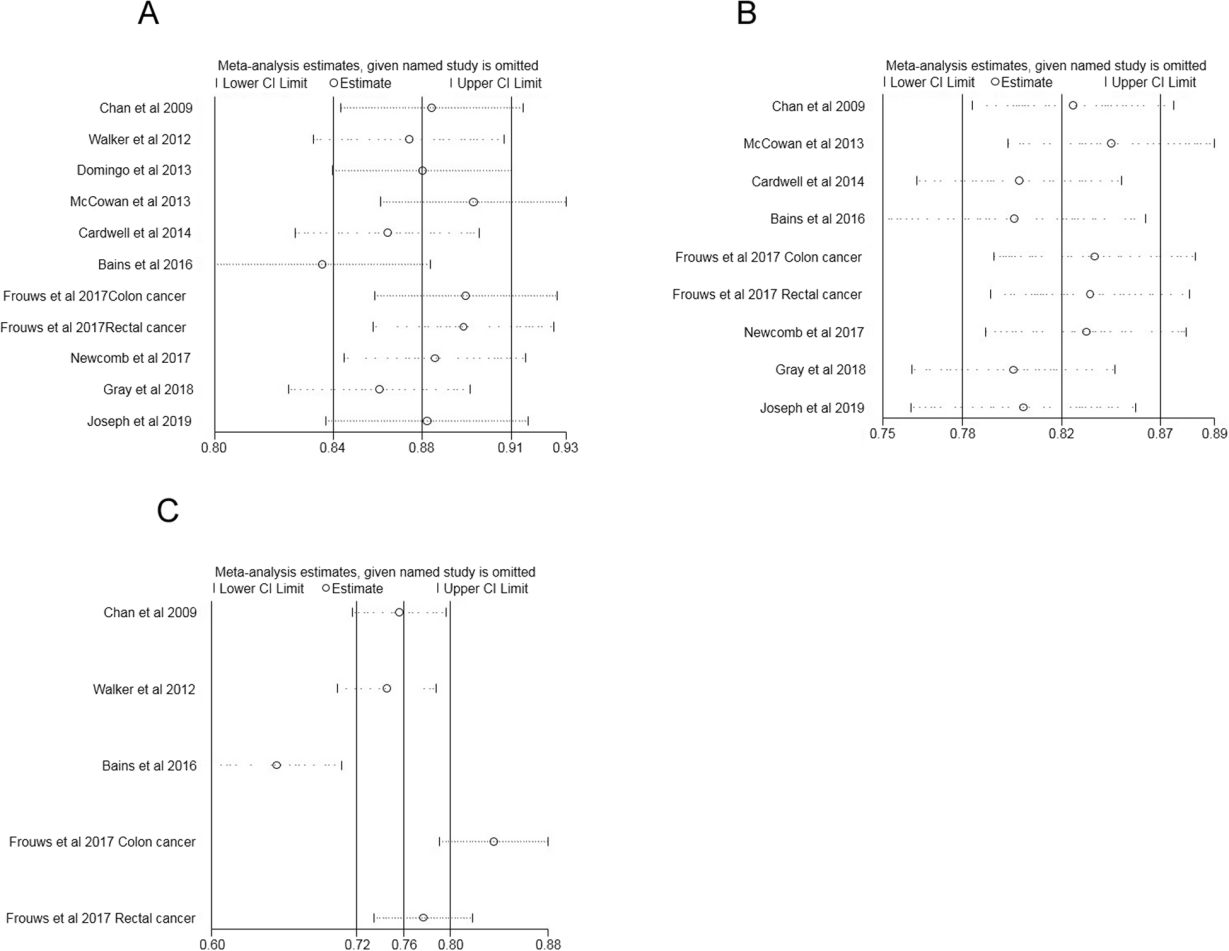
**a** Sensitivity analysis post-diagnosis aspirin use and overall survival for colorectal cancer. **b** Sensitivity analysis post-diagnosis aspirin use and cancer specific survival for colorectal cancer. **c** Sensitivity analysis both pre and post-diagnosis aspirin use and overall survival for colorectal cancer

Sensitivity analysis was performed to test the stability of the results by excluding each study successively. The results were not affected by sequential exclusion of any particular trial, except for one study (Bains et al., 2016). The detailed sensitivity analysis results are depicted in Fig. 6.

### Publication bias

In a meta-analysis with few studies (less than 10), the power of asymmetrical tests is too low to distinguish chance from real asymmetry. Because of the limited number of included studies, it was difficult to confirm the existence of publication bias in the current meta-analysis.

## Discussion

Aspirin is a nonselective cyclooxygenase inhibitor. Many studies [2–7] have observed that aspirin can improve the prognosis of digestive malignant tumors. However, there were some controversial issues in these studies, especially among those studies that focused on esophageal, gastric, and colorectal cancers with different gene mutation types, such as PIK3CA, that have survival benefits. This meta-analysis included 17 recent clinical studies with large sample sizes to investigate the effects of aspirin on the long-term survival of esophageal, gastric and colorectal cancers. Although the studies included were retrospective studies, they were of high quality and had large sample sizes. The results indicated that postdiagnosis aspirin use may improve OS and CSS in patients with colorectal cancer but not in patients with esophageal cancer or gastric cancer. Subgroup analysis indicated that postdiagnosis aspirin use could prolong the long-term survival of patients with PIK3CA gene mutations and high expression of PTGS2 (COX-2).

A Dutch cohort study [7] that involved 946 patients with esophageal cancer and 750 patients with gastric cancer demonstrated that postdiagnosis aspirin use significantly reduced mortality in esophageal cancer [HR = 0.42, 95% CI (0.30–0.57)] but failed to observe reduced mortality in gastric cancer [HR = 0.87, 95% CI 0.47–1.61]. Additionally, a British study [11] that included4654 patients with esophageal cancer and 3833 patients with gastric cancer observed that low-dose aspirin use did not reduce mortality in these patients. The present study also found that aspirin did not improve the overall survival rate of patients with esophageal and gastric cancer. Although the original studies had high quality and large sample sizes, more RCTs and evidence-based studies are needed because there are few studies that have focused on the long-term survival of patients with esophageal or gastric cancer.

Previous prospective studies [25, 26] have observed that aspirin can reduce colorectal adenomas and reduce the risk of colorectal adenomas recurrence. Most studies have found that aspirin should be used at least one year. The optimal dosage and duration is not consistent and large-scale prospective studies are still needed. This meta-analysis further supports that postdiagnosis aspirin use can improve the long-term survival of patients with colorectal cancer; however, prediagnosis aspirin use cannot improve the long-term survival of patients with colorectal cancer. As aspirin can lead to gastrointestinal bleeding and other side effects, it remains unclear whether low-dose aspirin can achieve adequate antitumor effects. Therefore, the long-term survival of patients with colorectal cancer needs to be evaluated with aspirin in the optimal dose and the best course of treatment. Moreover, side effects on the survival benefit of patients need to be investigated in the future. The daily dose of aspirin in the included observational studies was 75 mg–325 mg, and studies [27, 28] have shown that 81 mg aspirin is sufficient to inhibit rectal mucosal PGE2 production. The US Preventive Services Working Group [29] recommends 81 mg as a prescription dose for aspirin for the primary prevention of cardiovascular diseases and colorectal cancer. However, due to data limitations, a dose-response analysis between aspirin use and the long-term survival of patients with colorectal cancer was difficult to ascertain in the present study, and the optimal course of aspirin treatment needs to be investigated. We perform stratified analysis according to tumor stages. In patients with I-IV, aspirin may increase the overall survival (HR [0.88 (0.79, 0.98)]) and cancer-specific survival (HR [0.85 (0.74, 0.98)]) as shown in supplement Figure 3. We found that aspirin may increase CSS HR [0.73 (0.63, 0.85)] in stage II patients, but there was no survival benefit in other stages. Due to the limited literatures and high heterogeneity, more literatures need to be included for further analysis. Because the inclusion studies did not provide detailed information, it was impossible to conduct a subgroup analysis based on whether surgery or chemotherapy.

The mechanism of action of aspirin in the treatment of colorectal cancer is unclear. Some biomarkers can be used to predict the survival benefit of aspirin in colorectal cancer, including PTGS2 (COX-2) expression and the effects of the PIK3CA gene. The anti-inflammatory effects of aspirin are mediated through direct inhibition of COX-1 and COX- 2 [30–32]. PTGS2 (COX-2) promotes the inflammatory response and cell proliferation, and high expression of PTGS2 (COX-2) is associated with poor survival in patients with colorectal cancer [33, 34]. The up-regulation of PI3K enhances PTGS2 (COX-2) activity and prostaglandin synthesis and plays an important role in the signal transduction pathway of tumorigenesis [35, 36]. According to the subgroup analysis in our study, the effects of aspirin use on PIK3CA gene mutation and survival of patients with high expression of PTGS2 (COX-2) was different from that of patients with wildtype PIK3CA and PTGS2 (COX-2)-negative colorectal cancer. These findings provide a basis for the use of aspirin in patients with different types of mutations in colorectal cancer and the result can be used as a preliminary basis for further research.

Due to the bias of retrospective articles, it is necessary to perform randomised prospective studies to validate these data. At present, many clinical trials about aspirin and GI malignancies have not been completed. The ASAC trial (NCT03326791) are the first clinical interventional trial to assess the beneficial role of ASA in recurrence of CRC liver metastases and survival. Add-Aspirin (NCT02804815) aims to assess whether regular aspirin use after standard curative therapy can prevent recurrence and improve survival in individuals with non-metastatic common tumours. ASPIK French trial (NCT02945033) investigate Aspirin Versus Placebo in Resected Colon Cancer With PI3K Mutation Stage III or II High Risk. We also look forward to more prospective studies supporting the impact of aspirin on the prognosis of GI malignancies.

There were some limitations in this study. First, because the original studies were retrospective, there was some publication bias and selection bias. Second, due to the different definitions of aspirin use in the literature, the inclusion and exclusion criteria of the original studies were inconsistent; such differences may lead to deviations in the results. In addition, the number of studies involved was relatively small. Other potential confounding factors include the staging of tumors, whether surgery was performed, whether chemotherapy was performed, and the location of colorectal tumors. Because the included studies did not provide detailed information, it was impossible to conduct a subgroup analysis according to whether surgery, whether chemotherapy, the dosage, duration and reason for taking aspirin.

## Conclusion

In conclusion, based on the results of this study, aspirin can improve OS and CSS in patients with colorectal cancer after diagnosis, especially in those with PIK3CA gene mutations and high PTGS2 (COX-2) gene expression, but it cannot improve OS in patients with esophageal cancer and gastric cancer. The results provide a theoretical basis for the conductance of future RCTs. If RCTs can further confirm that aspirin can improve the long-term survival of patients with colorectal cancer, such therapies will have important clinical significance and socioeconomic value for patients with colorectal cancer because aspirin is inexpensive.

## Supplementary information

**Additional file 1: Figure 1A**. Flow diagram of the selection process of gastric cancer. **Figure 1B**. Flow diagram of the selection process of esophageal cancer. **Figure 1C**. Flow diagram of the selection process of colorectal cancer.

**Additional file 2: Supplementary file 2.** Post-diagnosis aspirin use and overall survival for esophageal cancer according to pathologic type. A subgroup analysis was conducted according to the pathologic type of esophageal cancer. The estimated pooled HRs showed no significant differences were seen between the two groups[HR = 1.05, 95%CI(0.92, 1.20)]of esophageal adenocarcinoma. The estimated pooled HRs showed no significant differences were seen between the two groups[HR = 0.89, 95%CI(0.74, 1.07)]of esophageal squamous cell carcinoma.

**Additional file 3: Figure 3A** aspirin use and overall survival for colorectal cancer according to tumor stage. **Figure 3B** aspirin use and cancer specific survival for colorectal cancer according to tumor stage.

## Data Availability

The data that support the findings of this study are available from the corresponding author upon reasonable request.

## Abbreviations

PPI: Proton Pump Inhibitor
RRs: Pooled risk ratios
OS: Overall survival
CSS: Cancer-specific survival
NOS: Newcastle–Ottawa Scale
RCTs: Random control trials
ASA: Acetylsalicylic acid
GI: Gastrointestinal
NSAIDs: Nonsteroidal Antiinflammatory Drugs
