# Supplementary figures and images for: Relationship between aspirin use of esophageal, gastric and colorectal cancer patient survival: a meta-analysis
Source: BMC Cancer. 2020 Jul 9;20:638. doi: 10.1186/s12885-020-07117-4 (PMC7350580; doi:10.1186/s12885-020-07117-4)

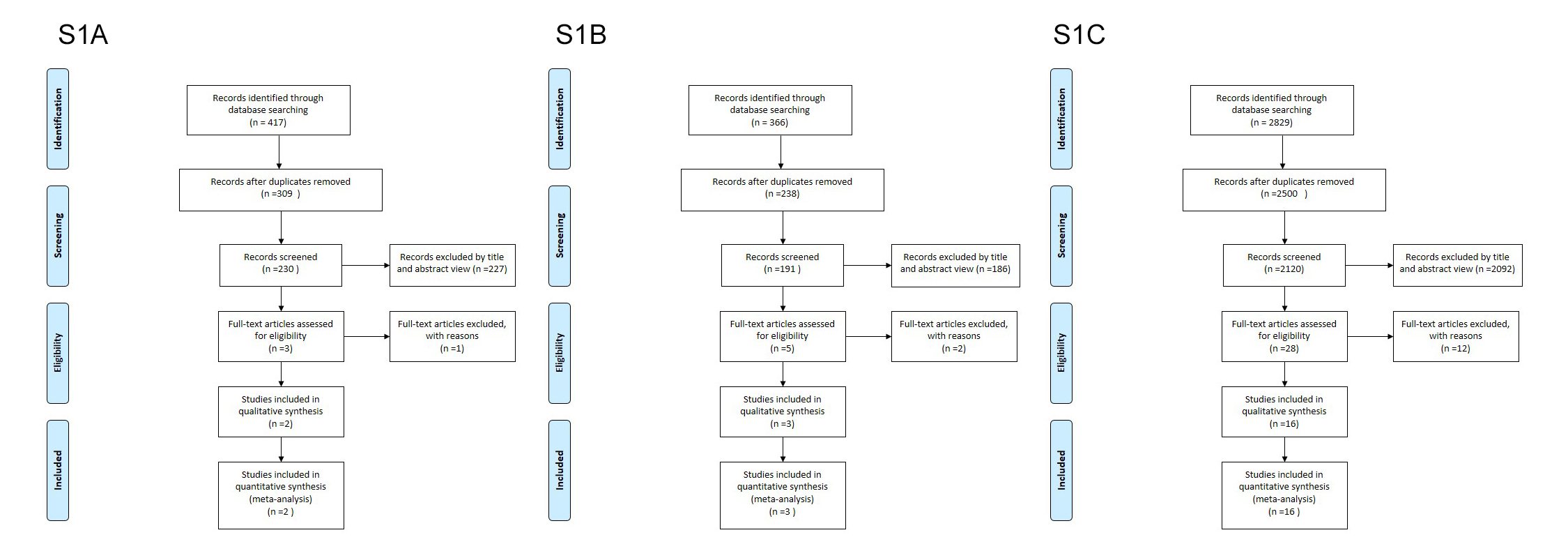

Supplement: Supplementary file 1 — Additional file 1: Figure 1A. Flow diagram of the selection process of gastric cancer. Figure 1B. Flow diagram of the selection process of esophageal cancer. Figure 1C. Flow diagram of the selection process of colorectal cancer. [file 12885_2020_7117_MOESM1_ESM.jpg]

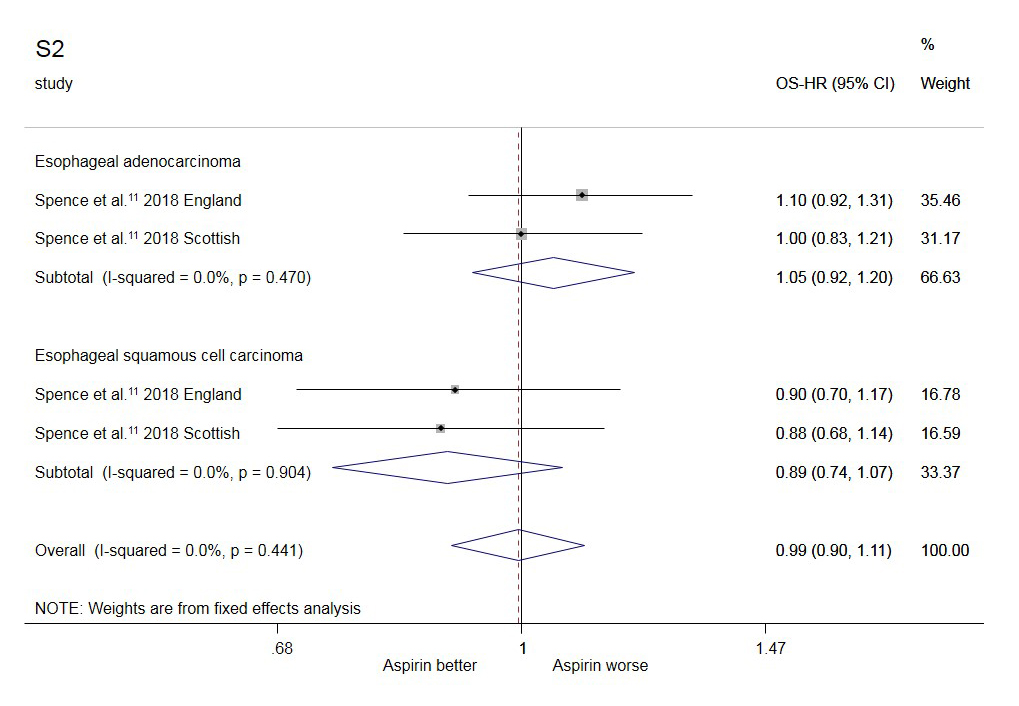

Supplement: Supplementary file 2 — Additional file 2: Supplementary file 2. Post-diagnosis aspirin use and overall survival for esophageal cancer according to pathologic type. A subgroup analysis was conducted according to the pathologic type of esophageal cancer. The estimated pooled HRs showed no significant differences were seen between the two groups[HR = 1.05, 95%CI(0.92, 1.20)]of esophageal adenocarcinoma. The estimated pooled HRs showed no significant differences were seen between the two groups[HR = 0.89, 95%CI(0.74, 1.07)]of esophageal squamous cell carcinoma. [file 12885_2020_7117_MOESM2_ESM.jpg]

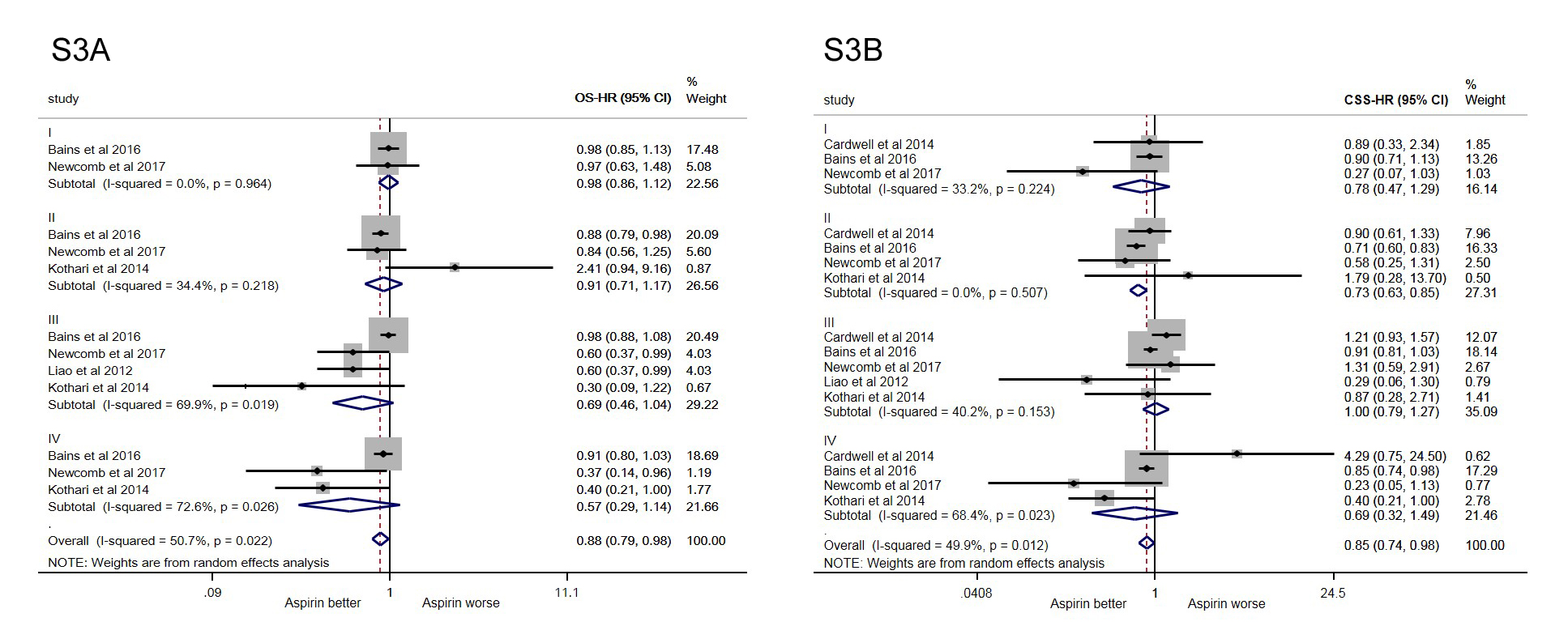

Supplement: Supplementary file 3 — Additional file 3: Figure 3A aspirin use and overall survival for colorectal cancer according to tumor stage. Figure 3B aspirin use and cancer specific survival for colorectal cancer according to tumor stage. [file 12885_2020_7117_MOESM3_ESM.jpg]
